# An Empirical Investigation of Factors Affecting Perceived Quality and Well-Being of Children Using an Online Child Helpline

**DOI:** 10.3390/ijerph16122193

**Published:** 2019-06-21

**Authors:** Willemijn van Dolen, Charles B. Weinberg

**Affiliations:** 1 Department of Marketing, University of Amsterdam Business School, Amsterdam 1018 TV, The Netherlands; 2 Sauder School of Business, University of British Columbia, Vancouver, BC V6T 1Z2, Canada; charles.weinberg@sauder.ubc.ca

**Keywords:** child helpline, quality, well-being, duration, chat share, emotional support, instrumental support

## Abstract

Child helplines provide free, accessible, and confidential support for children suffering from issues such as violence and abuse. Helplines lack the barriers often associated with the use of many other health services; and for many children, the helpline is the first point of contact with any kind of child protection and an important venue to go to in times of socio-economic distress. For instance, more children attempt to call the helpline in times of high unemployment, and relatively more of those conversations are about violence. Empirical evidence is scarce regarding how to implement online chat communication to improve quality and the child’s well-being. In this study, we focus on the impact of chat duration, number of words, and the type of support. The results show that for children seeking emotional support, a longer chat negatively influences the immediate well-being and the counsellor needs to listen (i.e., not type), as relatively more child words result in higher evaluations. We conclude that for emotional support, the counsellor should be prepared to listen carefully, but also manage the duration. However, for children chatting for instrumental support, the counsellor needs to type more to create positive perceptions of quality. Since the impact of chat share is different for children seeking emotional support (negative) versus instrumental support (positive), counsellors need to be sensitive to early indicators of the reason for the chat.

## 1. Introduction

Child helplines provide support for children about issues like violence at home, bullying, health, abuse, and sexuality, and the helplines also make referrals in case of an emergency. As such, child helplines, typically provided by non-profit organizations, help to fulfil the United Nations mandate that all children be heard. These helplines exist in 147 countries, receiving more than 24 million contact requests in 2017 [1]. 

Helplines lack the barriers often associated with the use of many other health services. That is, children have restricted access to more traditional healthcare services for which they need the consent and/or help of parents or other caregivers to get access. For children from low socio-economic positions in particular, financial barriers and non-financial barriers like transportation also come into play [2]. This is unfortunate, given that low socio-economic status is associated with poorer mental health and well-being of children and a stronger need for care [3]. Importantly, child helplines are free of charge and accessible for everyone with access to a (mobile) phone, as they can be contacted without the consent of parents, anonymously, from any location. As such, for many children, the helpline is the first point of contact with any kind of child protection and an important venue to go to in times of socio-economic distress. For instance, more children attempt to call the helpline in times of high unemployment [4]. 

Child helplines provide their services through different modalities like telephone, email, chat, and text-message (SMS). These modalities share the characteristics of being mediated, dialogue-based, and anonymous. However, whereas email, chat, and SMS are text-based, and therefore require literacy and typing skills, telephone is voice-oriented. Telephone and chat are synchronous and require presence (i.e., a child needs to be on the phone or in front of a computer), whereas email and SMS communication are asynchronous and do not require the constant presence of the child.

Our focus in this study is on the child’s evaluation of online chat sessions, as this modality is growing in popularity among children [1]. For chat to be successful in providing aid, children need to believe that their needs are being met. If so, they are more likely to tell others, either directly or through social media, about the availability of a specific helpline. This is particularly important for children who have limited economic means and are thus unlikely to have access to healthcare service alternatives. 

Research shows that the perceived quality of the service encounter with the helpline plays a critical role in determining children’s acceptance of online helplines as a viable source of assistance, as well as enabling these helplines to continue to have an impact on children’s well-being [5]. However, empirical evidence is scarce regarding how to design and manage chat communication to improve quality and the child’s well-being. Our research studies factors that drive the quality and effectiveness of online counselling in order to gain more insight into the benefits of online support [6,7,8]. We augment the literature on child well-being by extending the range of predictor variables studied to include two important but rarely investigated components of online chat: the chat share (in terms of number of words typed by the counsellor as compared to the child) and the duration (length of time that the chat lasts for) of the chat. Furthermore, since the reasons why children contact helplines differ, we examine the impact of support type sought (whether the child is seeking instrumental or emotional support) on the importance of chat share and duration on quality and the child’s sense of well-being.

A chat between a child and a counsellor is a two-way conversation. If one partner has a lot to communicate, he or she may act as the sender much more than the other partner. By being the sender for a greater extent, this partner exerts greater influence over the conversation. There is no literature suggesting how the role of the counsellor in relation to the child influences the child’s evaluation of the chat. Consequently, a prime focus of our research is on examining whether the counsellor’s relative word count affects the child’s perceived quality and immediate well-being after a single online chat. 

For most online child-helpline efforts, available resources are insufficient to cope with the number of chat requests received, and numerous chat attempts from children cannot get through due to lack of human capacity, infrastructure, and financial resources [1]. As a consequence, children often have to wait to begin their session and may even abandon their chat attempts, which is highly problematic given the serious nature of the chat content [9]. For those who do wait, the literature clearly indicates that longer waits lead to lower quality perceptions [10]. Additionally, counsellors experience stress when faced with the pressure during peak periods to handle more chats while also taking sufficient time to provide information and to listen to the child, or direction from management to keep the total chat duration low. If duration could be reduced without compromising outcomes, more children could be helped, and negative effects of waiting time and counsellor stress could be reduced.

Importantly, the impact of duration and relative word count on the quality and well-being of the child may vary depending on the type of support needed. Children look for support at helplines for different reasons [11]: some children need emotional support and sympathetic listening whereas others primarily look for practical information and advice, i.e., instrumental support. Our research studies how the relative number of words used by the counsellor and the duration of the chat influence children’s perceptions of quality and immediate well-being and how these effects vary by the type of support needed by the child. 

### 1.1. Types of Support

Children seek assistance from helplines for different types of support. Some children chat about problems that are characterized by high levels of emotional distress such as suicidal tendencies, violence, and depression. Often, the situation is critical and potentially harmful. Other children want help with more practical questions like problems with a teacher, contraception, and how to kiss. These problems require more informational support and advice, rather than emotional support. In other words, in some cases the cognitive need to be informed (the need to know and understand) is the primary concern, while the emotional need of the child (the need to feel heard and understood) is the chief concern in other instances. 

This is consistent with the literature, in which two types of support have received the most attention: emotional and instrumental support [12]. Emotional support focuses on understanding and encouragement. Instrumental support refers to activities like offering advice, information, and suggestions aimed at solving a problem [13]. Given the different needs of children, it would seem that the counsellor should assume a larger role as the sender in the conversation when instrumental support is sought. On the other hand, when the child is seeking emotional support, he or she wants to be heard and the counsellor should be prepared to listen, so the child should play the larger role. This means that the impact of the counsellor’s chat share (i.e., relative word count) on children’s perceived quality and immediate well-being would vary depending on the type of support the child seeks. 

### 1.2. Counsellor Chat Share

Counsellor chat share is defined as the number of words that the counsellor types divided by the total number of words of the chat; that is, how much the counsellor types relative to the child. When children chat to obtain emotional support, it involves issues that children often have trouble talking about. Children want to be heard, so the counsellor needs to listen (i.e., not type) during the chat [14]. When the counsellor types more, there is less room for the child to share, and the child might experience the given support negatively, with lower perceptions of quality. 

When children chat because they want advice and information from the counsellor (i.e., instrumental support), the counsellor needs to type to provide this information. In these cases, a counsellor who primarily listens might create uncomfortable feelings for the child because the child is more interested in appropriately detailed information from the counsellor rather than space to share problems extensively. Therefore, we hypothesize the following:

**Hypothesis** **1a:** *Counsellor chat share has a negative relationship with perceptions of quality for children chatting for emotional support.*

**Hypothesis** **1b:** *Counsellor chat share has a positive relationship with perceptions of quality for children chatting for instrumental support.*

For children who seek help because they want to share their story (emotional support), the act of typing their story should positively influence their immediate well-being. For a child, writing down his or her thoughts can provide a therapeutic effect in itself [14] and is associated with improvements in well-being and a reduction in distress [15,16]. This effect is stronger when writing about emotional experiences compared to writing about less stressful topics [15]. When the counsellor types more, the child gets relatively less opportunity to share, so the immediate well-being after the chat will be lower compared with children who shared more.

Children who initiate contact to get advice from the counsellor (i.e., instrumental support) are not necessarily chatting to find relief or insights through typing, but rather to get information. In this case, the soothing effect from expressing strong emotions is not expected when they primarily seek information and advice. Therefore, we hypothesize:

**Hypothesis** **2a:** *Counsellor chat share has a negative relationship with immediate well-being for children chatting for emotional support.*

**Hypothesis** **2b:** *Counsellor chat share has no impact on immediate well-being for children chatting for instrumental support.*

### 1.3. Duration

Another important factor that can potentially influence children’s evaluation of a helpline chat is the duration of the chat. In contrast to chat share, we do not expect an impact from chat duration on perceived quality. Duration neglect refers to the fact that the evaluation of experiences is not influenced by the passage of time; individuals tend to neglect duration when it is not salient and in single experiences without a standard for comparison [17]. Additionally, in the context of patient–doctor encounters, several studies have reported that duration of the encounter was not related to patient evaluations of the service [18]. As a result, we hypothesize:

**Hypothesis** **3:** *The duration of the chat has no effect on children’s perceptions of quality of the chat.*

Regarding the influence of duration on well-being, research finds a negative relation between the length of a visit to an online support community and well-being [6]. Children cannot concentrate too long [19]. This tendency might be even stronger in the context of a chat, as this involves more mental demand, can create communication ambiguity [20], and may cause higher levels of frustration [21]. Additionally, research finds that spending a relatively long time writing about problems becomes burdensome and creates negative feelings [22]. This latter effect may specifically apply to children chatting for emotional support. In the case of instrumental support, children might develop negative feelings if they must concentrate too long. That is, if the amount of information that needs to be processed exceeds a certain threshold, an individual’s cognitive capacity will likely be impacted in a negative way, consequently diminishing performance. Summarizing, duration has a negative effect on well-being regardless of the purpose of the chat that the child initiates. Therefore, we expect that:

**Hypothesis** **4:** *The duration of the chat has a negative relationship with a child’s immediate well-being after the chat.*

### 1.4. Consequences of Quality and Immediate Well-Being

It is well known in the service literature that quality positively influences customer satisfaction across a wide range of services, including healthcare [23]. Additionally, it is well known that satisfaction has a positive influence on willingness to recommend a service, including ones for which no fee is charged [24]. We empirically examine whether these findings hold for children who use chat helplines.

## 2. Materials and Methods

### 2.1. Data

**Figure 1:**
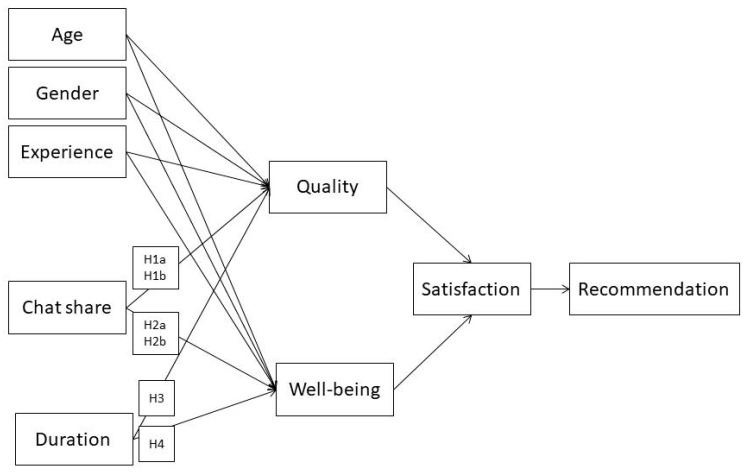
Hypothesized relationships.

The data for this study came from Kindertelefoon, which is the Netherlands’ largest child helpline. Between July 2010 and April 2011, children who contacted the helpline via online chat were asked to complete a survey regarding their experience with the service. When counsellors asked children to answer these questions at the end of the chat, they clearly emphasized that participation was voluntary and anonymous. In our study, we had completed questionnaires from 673 children. The mean age was 14 years (ranging from 8 to 18), and 85% were girls. For each chat, we had three types of data. First, the time spent waiting to start a chat, the duration of the chat, and a record of all words typed in a chat by the child and counsellor were all automatically collected by the helpline’s recording system (information from these chats was also used in other research by the authors [25], but the data on chat duration and word counts were not accessed for that earlier work). The chats were processed by a language processor to count the number of words typed by each conversation partner. Second, after every chat, the counsellors registered whether emotional or instrumental support was requested. Of the 673 chats, children requested instrumental support in 290 chats and emotional support in 383 chats. Third, information was obtained from questionnaires at the end of the chat, including evaluation of the chat (immediate well-being, perceived quality, satisfaction, and recommendation likelihood), chat experience with the helpline (41% first-time chatters), and age and gender. Figure 1 shows the hypothesized relationships.

### 2.2. Measurements

Chat share is the share of words typed by the counsellor as a fraction of the total number of words typed during the chat by the counsellor and child together. The duration was measured as the time from when the chat begins to the time when the chat ends. As a control variable, waiting time was included, which is the time from when the child first requests a chat until the chat begins.

The measures in the questionnaire came from previous studies and were adapted to the study context. Similar to earlier research on child helplines [5], single-item scales were used in order to increase the completion rate. Single-item scales are particularly useful when respondents are difficult to recruit and tend to have low response rates, such as children. Quality was measured on a 7-point Likert scale ranging from 1 (strongly disagree) to 7 (strongly agree), asking children to what extent they perceived that the child helpline delivers excellent service [26]. Satisfaction was measured by asking, “How satisfied are you with the child helpline?” [27] and answered using a 7-point Likert scale ranging from 1 (not satisfied at all) to 7 (completely satisfied). We adopted the well-being item used in earlier research [5] to “We would like to know how you feel at the moment” using a 7-point Likert scale ranging from 1 (you feel very bad right now) to 7 (you feel very good right now). Recommendation was measured with the item: “How likely is it that you will recommend the child helpline to a friend or peer?” In line with the literature [28], this item was measured on a 10-point Likert scale ranging from 0 (not at all likely) to 10 (extremely likely). 

## 3. Results

The correlation coefficients for the variables are presented in Table 1.

Summary statistics for the variables are in Table 2. 

We estimated the path model using multigroup structural equation modelling analysis within SPSS AMOS (IBM, Armonk, NY, USA). The data were organized into two groups, emotional and instrumental support. We constrained the coefficients to be the same for the models for emotional and instrumental support. A Chi-square differences test was used to compare the results of our constrained model with the unconstrained model, which indicated that the groups differed at the model level, i.e., the coefficients were dependent on the type of support (*χ*^2^ difference = 33.562, df = 16, *p* = 0.006). We tested the differences in coefficients for the two groups using z-scores. The results (Table 3) show that the model (unconstrained) fits the data well (*χ*^2^ = 201.206, df = 58; root mean square error of approximation (RMSEA) = 0.06; comparative fit index (CFI) = 0.85; incremental fit index = 0.85). 

A summary of the results for emotional and instrumental support chats can be found in Table 3. 

Chat share had a negative impact on the perceived quality for emotional support, confirming Hypothesis 1a. Additionally, Hypothesis 1b is supported, as chat share had a positive impact on the perceived quality for instrumental support. The difference between the two groups was significant (z = −4.772, *p* < 0.001). Chat share had a negative relationship with immediate well-being for emotional support and no impact on immediate well-being for instrumental support, offering evidence for Hypotheses 2a and 2b. This difference was significant between the two groups (z = −1.937, *p* < 0.054). The impact of duration on quality was nonsignificant for instrumental and emotional support (*p* > 0.10), supporting Hypothesis 3. By contrast, duration had a negative relationship with immediate well-being, but only for emotional support. Therefore, Hypothesis 4 is only partly supported. 

The path model further showed that for both types of chats, quality and well-being positively impacted satisfaction, which in turn positively influenced children’s willingness to recommend the helpline.

Age had a negative impact on quality for emotional support and on well-being for instrumental support. Gender and chat experience were not statistically significant. Waiting time had a marginally significant (*p* < 0.10) negative coefficient for quality.

## 4. Discussion

The primary goals of this research were to study the impact of counsellor chat share and duration of chat on children’s immediate well-being and their perceptions of quality, and to test whether this impact varied depending upon the type of support sought by the child. Our results suggest that, at least from the child’s perspective of the quality of the interaction, the role of the counsellor should vary by the type of support sought. To the best of our knowledge, this is the first empirical demonstration of such an effect.

We find, more specifically, that when children chat to obtain emotional support, the counsellor needs to listen (i.e., not type) during the chat, and relatively more child words result in higher evaluations. This is in line with the literature, which shows that individuals who generate detailed verbalizations about stressful issues exhibit greater health benefits like well-being [15]. However, when children chat for instrumental support, the counsellor needs to type more to provide this information.

Furthermore, our findings demonstrate that although duration does not influence quality, it may negatively impact children’s well-being, especially in cases of emotional support provision. This is in accord with earlier studies that found no [8] or a negative relation between duration and online interaction evaluations [6]. Overall, our finding with respect to duration suggests that counsellors might be able to shorten the length of the chat, possibly in turn reducing waiting time at peak periods. Our results do not allow us to make recommendations as to how short a chat should be, but rather to provide directional information. Moreover, the duration of a specific chat depends upon the child’s needs and the counsellor’s assessment of the issue and the child.

Combining our findings of duration and chat share, we come to the intriguing conclusion that for children who contact helplines to chat for emotional support, the counsellor should give the child the room to tell, but the chat sessions should not take too long; the counsellor should be prepared to listen carefully, but also manage the duration. Additionally, since the impact of chat share is different for children seeking emotional support (negative) versus instrumental support (positive), counsellors need to be sensitive to early indicators of the reason for the chat.

It is important to study child helplines, as this is often the first point of contact with any kind of child protection and an important venue for children to go to in times of socio-economic distress. It lacks the barriers that more traditional healthcare providers have, especially for children with lower socio-economic status. Additionally, many child helplines are open 365 days a year and for many hours per day [1], which makes them potentially a key resource for children who suffer from loneliness and look for emotional support during school holidays, a problem even more severe for low-income families [3]. Overall, it is important to study children, as they are in a phase marked with mental health problems, and recent research indicates that these problems have increased over the years, especially among girls [29]. As children are increasingly using the Internet to get social support [25] and health information [30] on their own, identifying the factors that lead to satisfaction and recommendations to their peers is relevant. Since personal recommendations from peers are likely to be particularly effective in convincing other children to use services like helplines, understanding the drivers of recommendations becomes crucial. Even when a service is not paid for, quality matters. 

### Limitations and Implications for Future Research

Like any other, this study is not without limitations. Regarding the impact of duration of the chat on children’s perceptions of the chat, content analysis could provide insight into whether duration is salient for children. Even if children are unaware of how long the conversation is taking, there still may be indications in the chats that the longer durations are generating negative emotions. More in-depth studies may also shed light on reasons why the impact of counsellor chat share differs depending on the type of support provided—a topic for future research. For instance, we argue it is likely that children chatting for emotional support benefit from writing about emotional topics, but the data from this study did not permit us to test this reasoning.

By design, child helplines are anonymous and do not allow for long-term follow-up on a child’s behaviour. Other types of counselling services may be able to shed light on the link between immediate responses to a counselling session and long-term health improvement, but such studies would be difficult to implement.

Further insights into chat behaviour can be facilitated by rapidly developing linguistic analysers that have the potential to determine the emotional content of online conversations. Child helplines and other online healthcare providers may be able to analyse their historical records of online chats to gain a better understanding of the factors affecting children’s quality perceptions. Field experiments based on analyses of historical data, like the present study, can be used to test the effect of major changes in policy. For example, if duration were reduced by either a quarter or a half, would there be a negative effect on perceptions of quality and/or a positive effect on well-being, at least in emotional chats? Such experiments could be conducted on a limited basis and would need to be monitored carefully.

Our study is limited to the data provided by the Dutch child helpline. However, hundreds of child helplines exist worldwide [1]. Future research with an international comparison of effects would be useful. Then, to generalize further, extensions to other services that provide support to children would be valuable. Finally, studies comparing the perceived quality and well-being for support provided via different channels, like face-to-face, email, and SMS, may shed light on how the medium shapes the communication.

## 5. Conclusions

We study online child helplines, an accessible form of healthcare provision for all children, that can be contacted free of charge, without the consent of parents, and anonymously. The nature of the help sought by children significantly impacts their evaluation of their service experience. We show that when children chat with the helpline to obtain emotional support, the counsellor needs to listen during the chat, and relatively more words by the child result in higher evaluations. When children chat for instrumental support, more counsellor words result in higher evaluations. Duration of the chat may negatively impact children’s well-being, especially in cases of emotional support provision. Counsellors might be able to shorten the length of the chat, possibly in turn reducing waiting time at peak periods. Combining our findings of duration and chat share, we conclude that for children who want to chat for emotional support, the counsellor should give the child the room to relate his or her concerns, but the chat sessions should not take too long.

## Figures and Tables

**Table 1:** Correlation table.

Variables	Age	Gender	Chat Experience	Waiting	Duration	Counsellor Share	Quality	Well-Being	Satisfaction
Age	1								
Gender	−0.112 *** (0.004)	1							
Chat Experience	−0.014 (0.712)	0.122 *** (0.002)	1						
Waiting Time	−0.06 (0.13)	0.068 * (0.072)	−0.036 (0.350)	1					
Duration	0.12 *** (0.002)	0.039 (0.313)	−0.104 *** (0.007)	0.089 ** (0.02)	1				
Counsellor Share	−0.13 *** (0.001)	0.008 (0.829)	0.074 * (0.054)	−0.053 (0.169)	−0.238 *** (0.000)	1			
Quality	−0.07 * (0.079)	−0.067 * (0.083)	−0.026 (0.495)	−0.091 ** (0.018)	−0.015 (0.700)	0.015 (0.690)	1		
Well-being	0.146 *** (0.000)	−0.014 (0.714)	0.019 (0.625)	−0.059 (0.125)	−0.103 *** (0.007)	0.004 (0.913)	0.372 *** (0.000)	1	
Satisfaction	−0.122 *** (0.001)	0.040 (0.304)	0.024 (0.541)	−0.064 * (0.095)	−0.027 (0.489)	0.019 (0.618)	0.714 *** (0.000)	0.464 *** (0.000)	1
Recommendation	0.150*** (0.000)	0.049 (0.203)	0.052 (0.176)	0.033 (0.389)	−0.003 (0.938)	0.083 ** (0.032)	0.519 *** (0.000)	0.327 *** (0.000)	0.556 *** (0.000)

Note: *** *p*-value < 0.01; ** *p*-value < 0.05; * *p*-value < 0.10.

**Table 2:** Summary statistics of sample (*n* = 673).

Variables	Statistics Mean (SD)
Waiting Time	5.37 min (3.98)
Number of Words Child	346.80 (251.59)
Number of Words Counsellor	401.18 (224.07)
Duration of Chat	25.70 min (15.2)
Counsellor Chat Share	0.56 (0.12)
Quality	5.86 (1.40)
Well-being	4.91 (1.68)
Satisfaction	5.84 (1.37)
Recommendation	8.81 (2.43)

**Table 3:** Summary of results for emotional and instrumental support chats.

Relationships			Emotional Standardized Coefficient	*p*-Value	Instrumental Standardized Coefficient	*p*-Value	z-Score
Control Variables							
Age	🡢	Quality	−0.096 *	0.054	−0.008	0.883	−0.956
Gender	🡢	Quality	−0.066	0.187	0.013	0.813	0.420
Chat Experience	🡢	Quality	−0.009	0.852	−0.046	0.414	0.341
Waiting Time	🡢	Quality	−0.087 *	0.081	−0.105 *	0.066	0.006
Age	🡢	Well-being	−0.082 *	0.077	−0.154 ***	0.005	1.247
Gender	🡢	Well-being	0.043	0.356	−0.068	0.214	2.379 **
Chat Experience	🡢	Well-being	0.036	0.433	0.001	0.990	0.437
Waiting Time	🡢	Well-being	−0.034	0.463	−0.012	0.826	−0.395
Independent Variables							
Counsellor Share	🡢	Quality	−0.144 ***	0.004	0.218 ***	0.000	−4.772 ***
Counsellor Share	🡢	Well-being	−0.098 **	0.037	0.038	0.497	−1.937 **
Duration	🡢	Quality	−0.013	0.789	0.051	0.370	−0.862
Duration	🡢	Well-being	−0.099 **	0.032	−0.058	0.283	−0.838
Quality	🡢	Satisfaction	0.606 ***	0.000	0.660 ***	0.000	0.304
Well-being	🡢	Satisfaction	0.270 ***	0.000	0.172 ***	0.000	2.002 **
Satisfaction	🡢	Recommend	0.567 ***	0.000	0.541 ***	0.000	0.292

Note: *** *p*-value < 0.01; ** *p*-value < 0.05; * *p*-value < 0.10. Note that z = −1.937 has a *p*-value of 0.054, which is coded as being significant when *p* < 0.05; 🡢 impact.
